# Clinical Predictors of COVID-19 Severity and Mortality: A Perspective

**DOI:** 10.3389/fcimb.2021.674277

**Published:** 2021-10-25

**Authors:** Jitender Sharma, Roopali Rajput, Manika Bhatia, Pooja Arora, Vikas Sood

**Affiliations:** ^1^ Department of Biochemistry, All India Institute of Medical Sciences, Bathinda, India; ^2^ Department of Biochemistry, School of Chemical and Life Sciences, Jamia Hamdard, New Delhi, India; ^3^ Department of Zoology, Hansraj College, University of Delhi, Delhi, India

**Keywords:** SARS-CoV-2, prognosis, biomarkers, risk factors, obesity, diabetes, radiological, sleep

## Abstract

The COVID-19 pandemic has caused huge socio-economic losses and continues to threat humans worldwide. With more than 4.5 million deaths and more than 221 million confirmed COVID-19 cases, the impact on physical, mental, social and economic resources is immeasurable. During any novel disease outbreak, one of the primary requirements for effective mitigation is the knowledge of clinical manifestations of the disease. However, in absence of any unique identifying characteristics, diagnosis/prognosis becomes difficult. It intensifies misperception and leads to delay in containment of disease spread. Numerous clinical research studies, systematic reviews and meta-analyses have generated considerable data on the same. However, identification of some of the distinct clinical signs and symptoms, disease progression biomarkers and the risk factors leading to adverse COVID-19 outcomes warrant in-depth understanding. In view of this, we assessed 20 systematic reviews and meta-analyses with an intent to understand some of the potential independent predictors/biomarkers/risk factors of COVID-19 severity and mortality.

## Introduction

Coronaviruses belong to Coronaviridae family of viruses. The degree of disease caused by coronaviruses can vary from mild like common cold to severe like severe acute respiratory syndrome (SARS) and the middle east respiratory syndrome (MERS). These viruses have been successful in crossing inter-species barriers. SARS-coronavirus jumped from civet cats to humans while MERS-coronavirus got transmitted to humans from camels (Woo et al., 2012). The recent emergence of the novel SARS Coronavirus 2 (SARS-CoV-2) is another incidence of zoonotic transmission of coronaviruses. As per the genomic sequence analysis, the source of novel SARS-CoV-2 is speculated to be a previously identified bat coronavirus strain RaTG13 (96.2- 97.41% identity match) (Shi, 2021; Malaiyan et al., 2021) or pangolin-CoV (91.02- 92.22% genomic identity match) (Zhang T. et al., 2020; Malaiyan et al., 2021). However, the origin of the SARS-CoV-2 is still unclear due to the lack of definitive evidence. Further investigations are being undertaken in this regard (WHO News release, 2021).

Since the first case reported late in 2019, SARS-CoV-2 has taken more than 4.5 million human lives (as of September 08, 2021) and continues to spread worldwide with more than 221 million confirmed cases (WHO, 2021). The case fatality rate of the disease caused by the SARS-CoV-2 (3.26- 4.16% in Latin America; 5.8% in the United States) (Undurraga et al., 2021; Loomba et al., 2021) is way less as compared to the previous coronavirus outbreaks (Zhu et al., 2020). Nevertheless, the fatality caused by Coronavirus Disease 2019 (COVID-19) has surpassed that of the SARS and MERS combined (Song et al., 2019). The COVID-19 pandemic has also resulted in huge economic losses (speculated to be trillions of dollars) around the world (Emem, 2020).

COVID-19 initially emerged as novel pneumonia of unknown etiology with majorly non-specific symptoms and quite quickly engulfed the entire globe. During the initial months of the pandemic, lack of specific diagnostic modalities, the variable intensity of the disease surveillance, changing case definitions, asymptomatic period of infection and overwhelmed health care facilities largely contributed to the rapid spread of the virus, resulting in the global outbreak. Also, the novel COVID-19 in a way bridged the gap between the developing and developed world, bringing all on the same footing. With more than 85 million confirmed cases, the Americas are the worst affected, followed by Europe (> 66 million), South-East Asia (> 41 million), the East Mediterranean region (> 15 million), Western Pacific (> 7 million) and Africa (> 5 million) (WHO, 2021). A major breakthrough in the current pandemic period witnessed rapid development and administration of different vaccines against COVID-19. However, despite the massive vaccine roll-out programs, the emergence of virus variants sustains the challenge of controlling the pandemic and continues to spread in its wild-type and mutant forms across the globe.

Since the onset of the disease, several groups have published various systematic reviews and meta-analyses that aim to shed light on the disease prognosis. However, the evidence was limited and the data were mostly heterogenous. Further, due to ever-changing viral dynamics, multiple new symptoms have been witnessed. With the generation of more data, it is expected that the analysis will continue with a focus on identifying unique clinical manifestations, laboratory findings, radiological investigations, and therapy that could correlate with varying degree of COVID-19 or adverse outcomes, and fatality. However, the studies published earlier have highlighted the significance of some important biomarkers and clinical features in diagnosis, prognosis and management of mild to severe COVID-19.

## Methodology

In the present work, we aim to identify key players of the disease and summarize important findings from already published studies on diverse clinical aspects of COVID-19. The search terms ‘COVID-19’, ‘SARS-CoV-2’, ‘clinical predictors’, ‘signs and symptoms’ were used individually or in appropriate combinations and only the ‘systematic reviews and/or meta-analysis’ articles that were published until February, 2021 were included for the present work. We carefully studied 20 systematic review/meta-analysis/meta-regression articles (**Table 1A**) that spanned the global population.

**Table 1A:** Overview of the methodology of the analyzed systematic reviews and meta-analysis in relation to severity, adverse prognosis and mortality of COVID-19.

S. No.	Reference	Date of publication (or acceptance for publication)	Methodology									
			Type of analysis	Diseases compared	Features analyzed	Data sources	Data set	Records screened	Records selected	Period (up to)	Total patients studied	Region of study
1.	Zhang JJY. et al., 2020	May 14, 2020	Systematic review, meta-analysis and meta-regression	COVID-19	Laboratory investigations as predictors of poor COVID-19 outcomes; and efficacy of therapies (involving experimental antiviral and immunomodulatory treatments)	Ovid MEDLINE, EMBASE, CENTRAL and PubMed	Heterogenous	1481	45	March 15, 2020	4203	China, Singapore, South Korea and Hong Kong
2.	Li et al., 2021	June 12, 2020	Systematic review and meta-analysis	COVID-19	Clinical features and outcome of severe and non -severe pneumonia patients	PubMed, EMBASE, Cochrane	Heterogenous	201	12	April 14, 2020	2445	China
3.	Földi et al., 2020	June 21, 2020	Systematic review, meta-analysis and meta-regression	COVID-19	Obesity as a risk factor	MEDLINE (via PubMed), EMBASE, CENTRAL, Scopus and Web of Science	Heterogenous	15168	24	May 11, 2020	Meta-analysis: 2,770 and 509 for ICU admission and IMV requirement, respectively Meta-regression: 2522	China, France, USA, Portugal, Netherlands, Italy and Qatar
4.	Lu et al., 2020	July 04, 2020	Systematic review and meta-analysis	COVID-19, SARS and MERS	Predictors of mortality	MEDLINE, Epistemonikos, COCHRANE, CKNI, WANFANG STATA and manual search	Heterogenous	712	28	April 11, 2020	16095 (COVID-19: 11818; SARS: 3292; MERS: 985)	COVID-19: China, Italy, South Korea and the United States
												SARS: Beijing, Guangdong, Shanxi, Hong Kong and Taiwan in China, and Toronto
												MERS: Saudi Arabia and South Korea
5.	Figliozzi et al., 2020	July 20, 2020	Systematic review and meta-analysis	COVID-19	Predictors of adverse prognosis	PubMed, MEDLINE, Scopus	Heterogenous	6843	49	April 24, 2020	20211	China, USA, France, Japan, Italy and Canada
6.	Henry et al., 2020	July 20, 2020	Systematic review and meta-analysis	COVID-19	Association of ‘at admission lymphopenia and neutrophilia’ with COVID-19 severity and mortality	PubMed, CNKI, CENTRAL	Heterogenous	53	22	May 06, 2020	4969	China, USA and Italy
7.	Shao et al., 2020	July 22, 2020	Systematic review and meta-analysis	COVID-19	Association of AKI with severe COVID-19 and related mortality	PubMed, Web of Science, Science Direct, medRxiv	Heterogenous	878	40	June 20, 2020	24527	China, South Korea, Korea, Spain, New York, Kuwait and Turkey
8.	Li et al., 2020	July 28, 2020	Systematic review and meta-analysis	COVID-19	Association of cardiac injury and severity and mortality	PubMed, EMBASE, Cochrane, CNKI, medRxiv, ChinaXiv	Heterogenous	1331	23	March 30, 2020	4631	China
9.	Ghahramani et al., 2020	August 03, 2020	Systematic review and meta-analysis	COVID-19	Laboratory features of severe and non-severe patients	PubMed, Web of Science, Science, EMBASE, Scopus	Heterogenous	1988	22	March 03, 2020	3396	China and Singapore
10.	Jutzeler et al., 2020	August 04, 2020	Systematic review and meta-analysis	COVID-19	Risk ratio of comorbidities, clinical features, laboratory parameters, imaging features, treatment and complications with morbidity and mortality	PubMed, Web of Science, EMBASE, Scopus, manual search	Heterogenous	2605	148	March 28, 2020	12149	China, Italy, USA, South Korea, Taiwan, Germany, France, Scotland, Japan, Vietnam, Canada, Singapore, Belgium, Finland, Russia, Spain and Sweden
11.	Lippi et al., 2020	August 25, 2020	Systematic review and meta-analysis	COVID-19	RDW as predictor of severity	MEDLINE, Web of Science, Science, Scopus	Heterogenous	13	3	July, 2020	11445	China, USA
12.	Moutchia et al., 2020	October 01, 2020	Systematic review and meta-analysis	COVID-19	Clinical laboratory parameters of severe or critical COVID-19	MEDLINE, EMBASE, Web of Science, CINAHL and Google Scholar databases	Heterogenous	1722	45	April, 18, 2020	9508	China, USA, France, Germany, Japan and Singapore
13.	Jahrami et al., 2021	October 13, 2020	Systematic review and meta-analysis	COVID-19	Impact of COVID-19 pandemic on quantity or quality of sleep	PubMed, MEDLINE, Web of Science, Science, Scopus and others	Heterogenous	371	44	July 05, 2020	54231	China, Iraq, Germany, India, Italy, France, Mexico, Spain, Bahrain, Greece, Australia and Canada
14.	Mudatsir et al., 2020	November 02, 2020	Systematic review and meta-analysis	COVID-19	Clinical manifestations and laboratory findings of mild to severe COVID-19	PubMed, EMBASE, Cochrane, Web of science	Heterogenous	39	19	April 05, 2020	3578	China (cities-Wuhan, Shenzen, Fuyang and Chongqing)
15.	Mesas et al., 2020	November 03, 2020	Systematic review and meta-analysis	COVID-19	Predictors of in-hospital mortality	PubMed, MEDLINE, Web of Science, Science, Scopus	Heterogenous	12254	60	May 17, 2020	51225	China, Italy, Israel, Pakistan, Brazil, Spain, UK, Switzerland, France, USA, South Korea and Iran
16.	Izcovich et al., 2020	November 17, 2020	Systematic review and meta-analysis	COVID-19	Prognostic factors for severity and mortality	PubMed, MEDLINE, EMBASE, CENTRAL	Heterogenous	569	207	April 28, 2020	57044	China, USA, Canada, Spain, France, Turkey, Korea, Japan, Italy, Germany, India and Singapore
17.	Del Zompo et al., 2020	December, 2020	Systematic review and meta-analysis	COVID-19	Prevalence of liver injury with COVID-19 severity and in-hospital fatality	PubMed, MEDLINE, PMC, EMBASE, Web of Science, clinical trial registries, publications from ArXiv, BioRxiv, Elsevier, MedRxiv, WHO sources and other databases searched for coronavirus family publications	Heterogenous	12484	36	August 03, 2020	20724	China, USA, Italy, South Korea, France and Germany
18.	Hannum et al., 2020	December 05, 2020	Systematic review and meta-analysis	COVID-19	Olfactory loss in COVID-19	PubMed, MEDLINE and Google Scholar	Heterogenous	43	34	April 30, 2020	19746	China, Italy, Sweden, Iran, Germany, Israel, Switzerland, UK, USA Taiwan, Korea, Belgium, Spain, France, Australia, Singapore and Iceland
19.	Israfil et al., 2021	January 11, 2021	Systematic review	COVID-19	Clinical characteristics	PubMed, Web of Science, Scopus, Science Direct, and Google Scholar	Heterogenous	557	34	May 07, 2020	10889	China, USA, Italy, Singapore, UK, France, Japan and Macau
20.	Poly et al., 2021	February 05, 2021	Systematic review and meta-analysis	COVID-19	Impact of obesity, associated comorbidities and other factors on risk of COVID-19 related mortality	PubMed, EMBASE, Google Scholar, Web of Science, and Scopus	Heterogenous	252	17	August 30, 2020	543399	China, Italy, Mexico, USA, France and UK

CENTRAL, Cochrane Central Register of Controlled Trials; CINAHL, Cumulative Index of Nursing and Allied Health Literature; CKNI, China National Knowledge Infrastructure; COVID-19, coronavirus disease 2019; EMBASE, Excerpta Medica database; MEDLINE, Medical Literature Analysis and Retrieval System Online; PMC, PubMed Central; UK, United Kingdom; USA, United States of America.

## Prognostic Factors Associated with Severe COVID-19

### Clinical Manifestations

Since the start of the pandemic, COVID-19 displayed a wide spectrum of clinical signs and symptoms, which included: fever, cough, sore throat, nasal congestion, sputum, headache, diarrhea, fatigue, dyspnea, chest tightness, myalgia, nausea, rhinorrhea, dizziness or confusion, hemoptysis, anorexia, vomiting, chest and abdominal pain (Huang et al., 2020; Jutzeler et al., 2020; Mudatsir et al., 2020). The eagerness to know any unique/distinct features was evident even in the layman. Fever, cough, fatigue, dyspnea (Figliozzi et al., 2020; Israfil et al., 2021) and a loss of sense of taste and smell (Hannum et al., 2020) remained some of the most experienced and identifying symptoms. In a systematic review involving more than 12000 patients, fever was the most common clinical manifestation in adults (78.5%), pregnant women (71.4%), pediatric and neonatal (53.1%) patients. Other important clinical signs and symptoms were cough (53.8%) and fatigue (25%) in adults, cough (41.4%) and myalgia (33.3%) in pregnant women and cough (47.9%) and sputum (27.5%) in children and neonates (Jutzeler et al., 2020). Only about 5% of patients were asymptomatic. Another meta-analysis, involving early data from 3578 patients, identified relation of dyspnea [odds ratio (OR)= 3.28, 95% confidence interval (CI) 2.09- 5.15], anorexia (OR= 1.83, 95% CI 1.00- 3.34), fatigue (OR= 2.00, 95% CI 1.25- 3.20) and dizziness (OR= 2.67, 95% CI 1.18- 6.01) with COVID-19 severity (Mudatsir et al., 2020). The vastly experienced COVID-19 symptoms, viz., fever, cough and breathing problem have been associated with problems in having sound sleep (Ferrando et al., 2016; Singh et al., 2020). An interesting systematic review and meta-analysis attempted to understand the impact of COVID-19 pandemic on quality or quantity of sleep under different study groups: COVID-19 patients, healthcare workers and the general population (Jahrami et al., 2021). As expected, about 75% of the COVID-19 patients had disturbed sleep, which was the highest prevalence among the different study groups (Jahrami et al., 2021). Physical pain or side-effects of the treatments were also speculated to impact the sound sleep in COVID-19 patients (Shi et al., 2020). These findings suggest that monitoring of sleep problems must not be ignored during COVID-19.

### Comorbidities as Risk Factors for Adverse Outcomes of COVID-19

In one of the early meta-analyses aimed at assessing the impact of comorbidities on the course and clinical outcome of COVID-19, it was found that about 31% of adult patients (2329/7608) had comorbidities, with hypertension being the most prevalent condition (20.93%, 1352/6460), followed by heart failure (10.5%, 37/354), diabetes mellitus (10.4%, 678/6535) and coronary heart disease (8.5%, 194/2388) (Jutzeler et al., 2020). These pre-existing comorbidities were found to be linked with the severity of COVID-19 (relative risk, RR= 2.11, p= 0.046) (Jutzeler et al., 2020). Also, hypertension (RR= 2.15, p< 0.001), diabetes (RR= 2.56, p= 0.005), any heart condition (RR= 4.09, p< 0.001) and chronic obstructive pulmonary disease (COPD) (RR= 5.10, p< 0.001) were associated with adverse disease outcome. In addition, disease severity was more in male (RR= 1.11, p= 0.039) and old age patients (standardized mean difference, SMD= 0.68, p< 0.001) (Jutzeler et al., 2020). The meta-analysis revealed that older age (SMD= 1.25, 95% CI 0.78– 1.72, p< 0.001), male gender (RR= 1.32, 95% CI 1.13–1.54, p= 0.005) and pre-existing comorbidities (RR= 1.69, 95% CI 1.48– 1.94, p< 0.001) were associated with less survival. Furthermore, mechanical ventilation was also more frequently required for treatment of non-survivors as compared to survivors (RR= 6.05, 95% CI 1.41– 26.05, p= 0.026); with more common administration of extracorporeal membrane oxygenation (RR= 4.39, 95% CI 1.64– 11.78, p= 0.014) in the non-survivors (Jutzeler et al., 2020). The risk of developing complications during the course of COVID-19 was higher in the non-survivors as compared to the survivors. The complications included, in particular, acute kidney injury (AKI) (RR= 20.77, 95% CI 2.43– 177.44, p= 0.017) and acute respiratory distress syndrome (ARDS) (RR= 4.24, 95% CI 1.30–13.83, p= 0.026) (Jutzeler et al., 2020).

Liver injury has been reported as another comorbidity being associated with the severity and in-hospital fatality of COVID-19 patients. In a meta-analysis of 20724 COVID-19 confirmed cases from 36 articles, pre-existing liver disease was present in up to 37.6% of cases (Del Zompo et al., 2020) at the time of hospital admission. The etiology of abnormal liver function was mentioned in only a few of the studies analyzed in the said meta-analysis. The authors recommended frequent testing of liver function test (LFT) markers as an additional tool for early stratification and monitoring of COVID-19 patients (Del Zompo et al., 2020). Further prospective cohort investigations are need-of-the-hour to validate the significance of LFT biochemistries in the management of COVID-19. Likewise, about 4.5% of COVID-19 patients displayed pre-known viral hepatitis in a study conducted by a different research group (Gu et al., 2020).

Another noteworthy comorbidity is AKI. In view of this, a systematic review and meta-analysis was conducted involving 24527 COVID-19 patients, where the overall rate of severe COVID-19 and COVID-19 related fatality was 26.4% and 20.3%, respectively (Shao et al., 2020). The robust meta-analysis revealed significant association of AKI with severity (OR= 8.11, 95% CI 5.01- 13.13, p< 0.00001) and fatality (OR= 14.63, 95% CI 9.94- 21.51, p< 0.00001) in COVID-19 patients. Prevalence of severe COVID-19 and fatality due to COVID-19 was considerably high (55.6% and 63.1% respectively, p< 0.01) in patients with AKI as compared to those without AKI (17.7% and 12.9% respectively) (Shao et al., 2020). Cardiac impairment was a significant factor associated with severe COVID-19 outcomes (OR= 3.15, 95% CI 2.26- 4.41) and fatality (OR= 1.95, 95% CI 1.08- 3.54) (Figliozzi et al., 2020; Li et al., 2020). Smoking (OR= 2.24, 95% CI 1.40- 3.58), history of diabetes mellitus (OR= 2.34, 95% CI 1.64- 3.33), COPD (OR= 2.63, 95% CI 1.55- 4.44) or hypertension (OR= 2.25, 95% CI 1.80- 2.82) contributed to progression to adverse COVID-19 (Figliozzi et al., 2020). Diabetes mellitus (OR= 1.74, 95% CI 1.22- 2.48), cardiovascular disease (OR= 1.95, 95% CI 1.08- 3.54), COPD (OR= 2.98, 95% CI 1.38-6.44), or cerebrovascular disease (OR= 2.93, 95% CI 1.84- 4.26) indicated high mortality risk (Figliozzi et al., 2020).

Apart from the above-mentioned somewhat obvious comorbidities, obesity emerged as another major condition that would worsen the outcomes in COVID-19 patients (Földi et al., 2020; Poly et al., 2021). A meta-analysis involving 2770 patients revealed that obesity was a significant risk factor associated with admission to critical care units (OR= 1.21, 95% CI 1.002- 1.46) (Földi et al., 2020). Also, the requirement of invasive mechanical ventilation (IMV) was more (up to 78%) for obese patients as analyzed in 509 subjects. A body-mass-index (BMI) of ≥ 25 was a significant risk factor for IMV requirement (OR= 2.63, 95% CI 1.64- 4.22) (Földi et al., 2020). Like obesity, psychiatric comorbidities (like anxiety and depression) must also be considered during COVID-19 management. Potential bi-directional associations between psychiatric comorbidities and sleep have been reported (Jahrami et al., 2021), amounting to sleep problems during COVID-19. This may impact the recovery from the disease.

### Biochemical Biomarkers as Independent Predictors of Severity, Adverse Prognosis or Mortality of COVID-19

Recent evidence highlighted the relevance of various biochemical tests as independent or combined correlates for the determination of severity, poor prognosis or mortality related to COVID-19. Clinical laboratory tests encompassing biochemical, hematological, inflammatory and coagulation parameters were considered useful to recognize severe or critical COVID-19. Additionally, these parameters also provided valuable clinical information for effective monitoring of the clinical course of COVID-19. As per findings of a large meta-analysis of 45 studies across 6 countries, neutrophilia (meta-median difference, MMD= 1.23 x 10^9^ cells/µl) and lymphopenia (MMD= -0.39 x 10^9^ cells/µl) were associated with critical COVID-19 (Moutchia et al., 2020). Similar findings were also reported in another meta-analysis comprising 4969 patients (Henry et al., 2020). In this meta-analysis, reduced lymphocyte count and increased neutrophil count at the time of admission were significantly associated with progression to severe disease (OR= 4.20, 95% CI 3.46- 5.09 and OR= 7.99, 95% CI 1.77- 36.14, respectively), and mortality (OR= 3.71, 95% CI, 1.63- 8.44 and OR= 7.87, 95% CI 1.75- 35.35, respectively) (Henry et al., 2020). Inflammatory markers, namely, C-reactive protein (CRP), Interleukin 6 (IL-6), and erythrocyte sedimentation rate (ESR) (MMD= 36.97 mg/l, 17.37 pg/ml, 21.93 mm/hr, respectively) were raised in severe COVID-19 cases (Moutchia et al., 2020). Biochemical indices like alanine aminotransferase (ALT), aspartate aminotransferase (AST), blood urea nitrogen (BUN), creatinine (MMD= 6.89 u/l, 11.96 u/l, 1.04 mmol/l, 4.87 µmol/l) were significantly elevated in severe or critical cases in comparison to non-severe COVID-19 patients (Moutchia et al., 2020). A meta-regression analysis observed that higher leukocyte counts (p< 0.0001), elevated levels of ALT (p= 0.024), AST (p= 0.0040), lactate dehydrogenase (LDH) (p< 0.0001) and raised procalcitonin (PCT) (p< 0.0001) were note-worthy predictors of admission to intensive care unit (Zhang JJY. et al., 2020). Further, the researchers found that elevated LDH (p< 0.0001) and high leukocyte counts (p= 0.0005) were significantly associated with COVID-19 led mortality. Other laboratory parameters that were found to be significantly associated with critical disease were myocardial biomarkers, Troponin I (MMD= 0.01 ng/ml), and creatine kinase-MB (CK-MB) (MMD= 1.46 u/l), tissue damage marker, LDH (MMD= 124.26 u/l), platelet count (MMD= -21.48 x 10^9^ cells/l) and D-dimer (MMD= 0.65 mg/ml) (Moutchia et al., 2020). These laboratory parameters indicated that innate immune response gets activated during COVID-19 as indicated by markedly raised neutrophil to lymphocyte ratio (NLR) and CRP. In contrast, adaptive immune response is unable to limit virus replication during severe COVID-19, as evidenced by reduced levels of lymphocytes and its subsets. Thus, routine testing for NLR, CRP, ESR, Troponin-I, BUN, creatinine, AST, ALT, CK-MB, LDH and D-dimer in severe COVID-19 is beneficial in monitoring clinical progression and can predict outcome of the disease. Anisocytosis, a condition that is characterized by heterogeneity in volumes of circulating red blood cells (RBCs), has also been linked to severe COVID-19. This low-cost parameter is expressed as RBC distribution width (RDW) and may be calculated as either RDW- standard deviation (SD) or coefficient of variation (CV). In this view, an analysis of RDW in 11445 COVID-19 patients was conducted and a 0.69% increase (95% CI 0.40- 0.98, p< 0.001) in absolute RDW-CV value of severe COVID-19 patients was found in comparison to mildly ill COVID-19 patients (Lippi et al., 2020). Hence, estimation of RDW may assist in risk stratification of adverse COVID-19 progression (Lippi et al., 2020).

Laboratory results were useful in differentiating severe from non-severe COVID-19 patients at the time of admission to the intensive care unit, as per the systematic review and meta−analysis conducted by Ghahramani et al. (Ghahramani et al., 2020). Results of routine tests like LFT, kidney function tests (KFT), glucose, albumin, electrolytes and complete blood count (CBC) were significantly altered in severe or critical COVID-19 patients belonging to the Asian population. In the same systematic review and meta-analysis, elevated PCT levels and higher neutrophil count were associated with bacterial co-infection in severe COVID-19 patients. Further, sensitivity analysis revealed significant differences in pooled effect size (p-ES) for NLR, lymphocyte to CRP ratio (LCR), PCT, AST, ALT, sodium, glucose, BUN, creatinine, ESR, myoglobin and D-dimer (Ghahramani et al., 2020). Laboratory parameters like decreased platelet count (p-ES= -1.7), low hemoglobin concentration (p-ES= -0.6), low albumin (p-ES= -3.1), raised IL-6 (p-ES = 2.4), elevated creatinine (p-ES = 2.4) and higher troponin-I (p-ES = 0.7) were markedly associated with in-hospital mortality (Mesas et al., 2020). As per another meta-analysis, low albumin levels (SMD= -1.13, 95% CI -1.41– -0.85, p< 0.001) and lymphocyte counts (SMD= -0.92, 95% CI -1.3– -0.55, p< 0.001) as well as high IL-6 levels (SMD= 1.21, 95% CI 0.93– 1.5, p< 0.001), leucocyte counts (SMD= 2.21, 95% CI 0.61– 3.64, p= 0.06), and prolonged prothrombin time (SMD= 7.99, 95% CI 4.64– 11.34, p< 0.01) were found to be linked with COVID-19 related mortality (Jutzeler et al., 2020). Hence, abnormal indices of the above-mentioned parameters could be prognostic markers of adverse COVID-19 outcomes.

In another large-scale analysis, including more than 57000 COVID-19 patients, 49 parameters were identified as high/moderate predictors of poor prognosis (Izcovich et al., 2020). The variable parameters included demographic factors: increasing age, male gender and smoking; comorbidities: diabetes, cerebrovascular disease, COPD, cardiovascular disease, cardiac arrhythmia, arterial hypertension, chronic kidney disease, cancer, dementia and dyslipidemia; physical examination factors: respiratory failure, fever, myalgia or arthralgia, fatigue, abdominal pain, tachycardia, hypoxemia, dyspnea, anorexia, tachypnoea, low blood pressure, hemoptysis; laboratory assessments: elevated PCT, myocardial injury markers, increased WBC counts, elevated blood lactate, reduced lymphocyte count, reduced platelet count, increased neutrophil count, raised plasma creatinine, elevated D-dimer, raised LDH, elevated CRP, raised AST levels, decreased albumin, elevated IL-6 levels, raised B-type natriuretic peptide (BNP), elevated BUN, raised ESR, elevated CK and raised bilirubin; radiological factors: pleural effusion and consolidative infiltrate; and high sequential organ failure assessment (SOFA) score (**Tables 1B**, **2**) (Izcovich et al., 2020).

**Table 1B:** Summary of the major outcomes of the analyzed systematic reviews and meta-analyses.

S. No.	Reference	Outcome						Conclusion
		Demographics	Signs and symptoms	Comorbidities	Laboratory findings	Radiological (CT scan) findings	Therapies	
1.	Zhang JJY. et al., 2020	• Higher proportion of males (66.5%) vs. females (33.5%) suffered from COVID-19; • Pooled mean age: 45 years (95% CI, 35.5– 54.5 years; • ICU admission: 10.9%, patients analyzed= 2153; • Mortality: 4.3%, patients analyzed = 2921.	• Most common: fever (80.5%, patients analyzed= 3934), cough (58.3%, patients analyzed= 3718) and dyspnea; • (23.8%, patients analyzed= 2992); • Fever definition as: ≥ 37.3°C (7 studies) or ≥ 37.5°C (2 studies); • Pooled mean incubation period: 6.1 days (95% CI 5.0– 7.3 days); • Pooled mean time from onset of symptoms to hospital admission: 7.2 days (95% CI 5.5– 8.9 days).	• Most common: Hypertension (16.4%, patients analyzed= 2928), cardiovascular diseases (12.1%, patients analyzed= 1498) and diabetes mellitus (9.8%, patients analyzed= 3060).	• Most common: Increased levels of CRP (59.4%) and lactate dehydrogenase (LDH) (51.7%), low levels of albumin (58.6%) and lymphopenia (47.7%).	• Most common: Bilateral infiltrates (80.8%), ground-glass opacities (73.0%, patients analyzed= 2618), interlobular septal thickening (46.3%, patients analyzed= 522), subpleural lines (45.5%, patients analyzed= 245), and consolidation (41.6%, patients analyzed= 2395).	• Antivirals- Combinations of oseltamivir, ganciclovir, lopinavir, ritonavir, ribavirin, arbidol; • Antibiotics- Moxifloxacin, ceftriaxone, azithromycin.	• High counts of leukocytes, high levels of ALT, AST, LDH, and PCT are important laboratory markers that are associated with ICU admission, mortality and ARDS; • Use of corticosteroids is significantly associated with higher proportion of patients with ARDS; • Use of lopinavir and ritonavir is not distinctly related to lowering mortality due to COVID-19; • Further prospective studies are necessary to validate the findings.
2.	Li et al., 2021	• COVID-19 severity not significantly linked with gender (OR= 1.14, 95% CI 0.91- 1.43, I^2 ^= 0.0%, p= 0.267) or Wuhan exposure history (OR= 0.92, 95% CI 0.53-1.59, I^2 ^= 0.0%, p= 0.764); • Smoking significantly associated with severe COVID-19 (OR = 1.70, 95% CI: 1.20-2.41, I^2 ^= 43.4%, p= 0.003).	• Fever (OR= 1.67, 95% CI 1.15- 2.42, p= 0.007, I^2 ^= 38.8%) and dyspnea (OR= 4.17, 95% CI 2.04- 8.53, p< 0.001, I^2 ^= 71.3%) related to severe COVID-19.	• Severity or ICU admission related to diabetes (OR= 3.17, 95% CI 2.26- 4.45, p< 0.001, I^2 ^= 35.3%), COPD (OR= 5.08, 95% CI 2.68- 9.63, p< 0.001, I^2 ^= 0.0%), coronary heart disease (OR= 2.66, 95% CI 1.71-4.15, p< 0.001, I^2 ^= 0.0%), hypertension (OR= 2.40, 95% CI 1.47-3.90, p< 0.001, I^2 ^= 51.5%), cerebrovascular diseases (OR= 2.68, 95% CI 1.29- 5.57, p= 0.008, I^2 ^= 41.8%), and malignancy (OR= 2.21, 95% CI 1.04-4.72, p= 0.040, I^2 ^= 0.0%).	• Severity indicators: • Elevated leucocyte counts (OR= 3.46, 95% CI 1.06- 11.28, p= 0.040, I^2 ^= 75.1%), PCT (OR= 6.69, 95% CI 3.99- 11.20, p≤ 0.001, I^2 ^= 13.6%), CRP (OR= 4.02, 95% CI 2.80- 5.79, p≤ 0.001, I^2 ^= 11.1%), LDH (OR= 3.36, 95% CI 2.46- 4.58, p< 0.001, I^2 ^= 48.3%), AST (OR= 3.26, 95% CI 2.40- 4.42, p< 0.001, I^2 ^= 5.3%), ALT (OR= 1.95, 95% CI 1.35- 2.80, p< 0.001, I^2 ^= 39.6%), Creatinine (OR= 2.14, 95% CI 1.14- 4.01, p= 0.018, I^2 ^= 0.0%), CK (OR= 2.45, 95% CI 1.69- 3.55, p< 0.001, I^2 ^= 46.7%); • Decreased platelets (OR= 2.82, 95% CI 2.07- 3.83, p< 0.001, I^2 ^= 0.0%) and lymphocytes (OR= 4.60, 95% CI 3.25- 6.51, p< 0.001, I^2 ^= 0.0%).	NA	• Severe COVID-19 or ICU admitted patients required more frequent use of: Antibiotics (OR= 3.58, 95% CI 1.29- 9.87, p= 0.014, I^2 ^= 84.1%), antivirals (OR= 1.79, 95% CI 1.35- 2.38, p< 0.001, I^2 ^= 0.0%), systemic corticosteroids (OR= 5.46, 95% CI 4.17- 7.14, p< 0.001, I^2 ^= 0.0%), mechanical ventilation including invasive and non-invasive ventilation (OR= 171.72, 95% CI 27.38- 1,077.21, p< 0.001, I^2 ^= 73.2%), ECMO (OR= 29.36, 95% CI 5.36- 160.68, p< 0.001, I^2 ^= 0.0%) and continuous renal replacement therapy (OR= 25.45, 95% CI 6.97- 92.89, p< 0.001, I^2 ^= 0.0%).	• Significant differences in outcome of severe and non-severe pneumonia in terms of discharge and death were observed.
3.	Földi et al., 2020	• A range of 9% to 43% (2,770 patients from 6 studies) patients required ICU admission; • A range of 58% to 78% (509 patients from 5 studies) required IMV.	NA	• Obesity significantly associated with higher risk for ICU admission (OR= 1.21, 95% CI 1.002- 1.46, I^2 ^= 0.0%); • Obesity associated with higher risk for IMV (OR= 2.05, 95% CI 1.16- 3.64, I^2 ^= 34.86%); • 6 times higher risk for ICU admission in patients with BMI≥ 35 as compared to BMI< 25 (OR= 6.16, 95% CI 1.42- 26.66).	NA	NA	• Significantly higher likelihood of IMV requirement in patients with BMI≥ 25 as compared to BMI< 25 (OR= 2.63, 95% CI 1.64- 4.22, I^2 ^= 0.0%).	• Obesity may serve as important clinical predictor of risk gradation for COVID-19, and related ICU admission, especially IMV requirement; • Higher BMI ranges carried significantly higher risk for IMV in contrast to lower BMI ranges; • Careful monitoring of obese patients is necessary to better manage COVID-19; • Early escalation of therapy may be needed in such patients to dodge unfavorable clinical outcomes; • High recommendation to improve guidelines for patients with obesity owing to the returning pandemic waves.
4.	Lu et al., 2020	• Higher mortality rates in elderly (total OR= 7.86, 95% CI 5.46– 11.29; COVID-19: OR= 6.45, 95% CI 3.86– 10.77; SARS: OR= 11.97, 95% CI 8.82– 16.24; MERS: OR= 7.02, 95% CI 4.59–10.73); • Among the non-survivors, higher mortality rate observed in males (total OR= 1.82, 95% CI 1.56– 2.13; COVID-19: OR= 1.96, 95% CI 1.43– 2.69; SARS: OR= 1.81, 95% CI 1.43– 2.30; MERS: OR= 1.74, 95% CI 1.32- 2.30). Higher mortality rate observed in patients with comorbidities (total OR= 4.41, • 95% CI 3.18– 6.12; COVID-19: OR= 3.50, 95% CI 2.35– 5.20; SARS: • OR= 6.47, 95% CI 4.93– 8.50; MERS: OR= 3.08, 95% CI 0.35– 27.01.	• Respiratory rate was a sensitive indicator of mortality for COVID-19 (OR= 4.90, 95% CI 1.08– 22.24) and SARS (OR= 8.88, 95% CI 5.64– 13.97).	• Chronic lung disease, hypertension, diabetes, increasing age and male gender.	• Predictors of mortality for COVID-19 patients: Lower platelet count (OR= 0.33, 95% CI 0.24– 0.44), lower lymphocyte counts (OR= 0.21, 95% CI 0.12– 0.38), higher neutrophil (OR= 17.56, 95% CI 10.67– 28.90), raised WBC count (OR= 9.13, 95% CI 5.71– 14.59), decreased albumin levels (OR= 0.11, 95% CI 0.06– 0.19), raised LDH (OR= 37.52, 95% CI 24.68– 57.03), elevated CRP (OR= 12.11, 95% CI 5.24– 27.98) and elevated BUN (OR= 8.49, 95% CI 5.81– 12.40); • Mortality indicators for all 3 coronavirus diseases, i.e., SARS, MERS and COVID-19: LDH, neutrophils, CRP, BUN and albumin; • Higher variation among laboratory parameters in COVID-19 as compared to SARS and MERS.	• Similar pulmonary consolidation and bilateral GGO observed in SARS, MERS and COVID-19; • Majority of the COVID-19 patients with above-said abnormal imaging features died (consolidation: OR= 3.26, 95% CI 1.16– 9.13; GGO: OR= 1.45, 95% CI 0.47– 4.49).	NA	• Mortality indicators for COVID-19 are similar to SARS and MERS.
5.	Figliozzi et al., 2020	• Patients aged above 70 years had 13-fold higher odds of death than younger patients (OR= 13.19, 95% CI 7.72- 22.55); • Males had higher risk of death (OR= 1.71, 95% CI 1.39- 2.09, p< 0.001).	• Non-productive cough and fever.	• Diabetes mellitus (OR= 1.74, 95% CI 1.22-2.48, n = 13), history of CVD (OR= 1.95, 95% CI 1.08- 3.54, n = 7) or cerebrovascular disease (OR= 2.93, 95% CI 1.84- 4.26, n= 5), hypertension (OR= 2.71, 95% CI 1.99- 3.69, n = 15) and COPD (OR= 2.98, 95% CI 1.38-6.44, n= 8) associated with higher risk of mortality; • Progressing age associated with worse prognosis (p- OR= 1.027 per year, 95% CI 1.00- 1.06, p= 0.069); • Hypertension identified as an overall link between increasing age and worse prognosis; • Male gender associated with higher risk of mortality; • Smoking not a predictor of mortality (OR= 3.14, 95% CI 0.48-20.56, n= 4), but only associated with greater likelihood of composite adverse outcome (OR= 2.24 per comparison to non-smokers, 95% CI 1.40-3.58, p= 0.003, n= 11).	• Elevated CRP levels, D-dimer levels, and lymphocytopenia.	NA	• During acute phase: steroids, antibiotics and antivirals.	• High odds of mortality indicated by various comorbidities, laboratory findings and increasing age.
6.	Henry et al., 2020	• Severe lymphopenia: Number of total patients ranged from 12 (6 severe) to 1099 (153 severe); females: 15% to 50%; age range of severe cases: 25 to 87 years; • Fatal lymphopenia: Number of total patients ranged from 108 (96 non-survivors) to 274 (113 non-survivors); females: 27% to 41%; Age range of fatal cases: 51 to 84 years; • Severe neutrophilia: Total patients ranged from 12 (6 severe) to 548 (267 severe); Females: 34.4% to 50%; Age range of severe cases: 38 to 72 years; • Fatal neutrophilia (2 studies): Total patients 144 (70 severe) and 274 (113 severe); Females: 35.7% and 27%; Age range of fatal cases: 62 to 84 years.	NA	NA	• Admission lymphopenia significantly indicated more than 4-fold increased risk of developing severe COVID-19 (OR= 4.20, 95% CI 3.46- 5.09, p< 0.001; I^2 ^= 0.0%) and in-hospital mortality (OR= 3.71, 95% CI 1.63- 8.44, p= 0.002; I^2 ^= 88.4%); • Admission neutrophilia significantly linked to 8-fold increased odd of developing severe COVID-19 (OR= 7.99, 95% CI 1.77- 36.14, p=0.007, I^2 ^= 75.9) and mortality (OR= 7.87, 95% CI 1.75- 35.35, p= 0.007, I^2 ^= 89.3).	NA	NA	• Lymphopenia and neutrophilia at first visit should be included in risk stratification models; These are independent risk factors for adverse outcome.
7.	Shao et al., 2020	• Number of patients ranged from 41 to 5700; Mean/median age: 45.4 ± 17.2 to 69 years; Males: 38.8% to 73%.	NA	• AKI in 10% (95% CI 8– 13) COVID-19 patients (with statistical heterogeneity among the studies analyzed, I^2 ^= 98%); • AKI significantly associated with high mortality (OR = 14.63, 95% CI 9.94–21.51, p< 0.00001, I^2 ^= 77%, p< 0.01).	• Higher SCr levels (MD= 20.19 μmol/l, 95% CI 14.96– 25.42, p< 0.00001, I^2 ^= 55%, Cochran’s Q, p= 0.03); • Higher BUN levels associated with severity and mortality.	NA	NA	• AKI significantly associated with fatality in COVID-19 patients; • Kidney damage monitoring crucial during early stage of COVID-19.
8.	Li et al., 2020	• Total subjects analyzed: 4631; Among the studies analyzed sample size ranged from 41 to 1099 and was over 100 in 16 studies; Males: 42.5% to 73.2%; Mean/median age: 43.1 to 62 years.	NA	NA	• Elevated TnI levels associated with severity (64.5%), ICU admission (56%) and mortality (59.3%); • Mean NT-proBNP levels significantly higher in patients with elevated TnI levels (SMD= 1.63, 95% CI 1.02- 2.23, p< 0.001; I^2 ^= 86.6%) than the ones with non-elevated TnI; • Higher mean CK levels significantly associated with severity/ICU admission (SMD= 0.39, 95% CI 0.11- 0.67, p= 0.006; I^2 ^= 69.0%); • Elevated CK-MB levels more frequent in severe COVID-19/ICU-admitted patients (45.7%); • Higher CK-MB levels associated with higher risk of severe COVID-19 or ICU admission (RR= 3.24, 95% CI 1.66- 6.34, p= 0.001, I^2 ^= 79.8%); • Increased LDH levels in 60.1% of severe or ICU-admitted patients; • Higher LDH levels associated with increased risk of severity or ICU admission (RR= 2.20, 95% CI 1.55- 3.12, p< 0.001, I^2 ^= 79.7%); Elevated levels of IL-6 significantly associated with severity or ICU admission (SMD= 0.54, 95% CI 0.27- 0.81, p< 0.001, I^2 ^= 0.0%) and mortality (SMD= 1.28, 95% CI 1.00- 1.57, p< 0.001, I^2 ^= 13.7%); • Emerging arrythmia linked to higher risk of severity or ICU admission (RR= 13.09, 95% CI 7.00- 24.47, p< 0.001, I^2 ^= 42.0%).	NA	NA	• Combination of cardiac examination and biomarkers can improve accuracy of cardiac injury assessment; • Careful monitoring of cardiac injury biomarkers during acute phase of COVID-19 is recommended.
9.	Ghahramani et al., 2020	• A total of 3396 (range 12 to 1099) patients analyzed; • Severe: 720 and non-severe: 2676.	NA	NA	• Decreased levels of sodium, lymphocytes, monocytes, eosinophil, hemoglobin and platelets, albumin; • Increased levels of ALT, AST, total bilirubin, BUN, creatinine, CRP, LDH, PCT, ESR and glucose.	NA	NA	• Results of CBC, LFT, KFT, inflammatory markers, glucose and electrolytes significantly varied between severe and non-severe patients; • Further studies in other populations are recommended.
10.	Jutzeler et al., 2020	• Higher proportion of males vs. females suffered from COVID-19 across all single studies analyzed.	• Most common in **adult** patients: Fever (78.5%, 6955/8859), cough (53.8%, 4778/8885) and fatigue (25%, 1996/7980); • Most common in **pregnant women** patients: Fever (71.4%, 25/35), cough (41.4%, 12/29) and myalgia (33.3%, 3/9); • Most common in **pediatric and neonatal** patients: Fever (53.1%, 170/320), cough (47.9%, 149/311) and sputum (27.5%, 14/51 patients); • **Asymptomatic** patients: Overall (7.8%, 297/3′822) including 5.4% adult (148/2749) patients, 14% (149/1054) children and neonates.	• Most common in **adult** patients: Hypertension (20.93%, 1352/6460), heart failure (10.5%, 37/354), diabetes mellitus (10.4%, 678/6535), coronary heart disease (8.5%, 194/2388); • Only 5 **pregnant women** patients had comorbidities, of which 2 were unidentified, while the other 3 were: Hypothyroidism, allergies or influenza; • None in **pediatric and neonatal** patients, except 2 children.	• **Adults**: Elevated levels of IL-6 [22 pg/ml (4.68–51.8)], erythrocyte sedimentation rate [32.5 mm/h (17.3–53.8)], D-dimer [0.5 μg/m (0.3–1.08)], fibrinogen [4.5 g/l (3.66–5.1)], and LDH [213 u/l (173–268)]; • **Pregnant women**: Increased levels of CRP [19.25 mg/l (12.35–25.7)], procalcitonin (0.187 ng/ml), neutrophil count (9.14 × 10^9^/l) and lactate dehydrogenase (544 u/l); • **Pediatrics and neonates**: No generalized conclusions can be made as the normative values are age-dependent within this age-group.	• **Adults**: Pneumonia (unilateral or bilateral, 83.6%, 6620/7917), including air bronchogram (50.5%, 264/523) and GGO with consolidation (47.4%, 153/323) and without (43.8%, 2446/5591); • **Pregnant women**: Pneumonia (unilateral or bilateral, 88.6%, 31/35), GGO (85.3%, 29/34) and consolidation (50%, 8/16); • **Pediatrics and neonates**: Pneumonia (65%, 194/298), GGO (38.9%, 108/278) and local patchy shadowing (23.3%, 52/223).	• **Adults**: Antivirals (73.8%, 4475/6068), oxygen therapy (69.4%, 1300/1872) and antibiotics (52.2%, 2518/4825); **Pregnant women**: Antibiotics (100%, 14/14), antivirals (78.6%, 11/14) and oxygen therapy (high flow nasal cannula; 25%, 3/12); • **Pediatrics and neonates**: Antibiotics (72.1%, 31/43), oxygen therapy (high flow nasal cannula; 55.6%, 5/9) and alpha interferon aerosol inhalation therapy (59.6%, 31/52).	• Clinical signs and imaging features comparable between survivors and non-survivors. Pre-existing comorbidities associated with increased disease severity; • Abnormal laboratory tested blood parameters are associated with disease severity.
11.	Lippi et al., 2020	• Only 3 studies with total 11445 (161 to 11095 range) samples and 2654 (23.2%) severe cases; Females: 41.3% to 48.9%; Mean age: 39 ± 13 to 65 ± 7 years.	NA	NA	• RDW-CV values are raised in severe illness; • Increased RDW at admission carried 2.5-fold risk of in-hospital mortality; • Incremental RDW values in hospitalized patients associated with increased mortality	NA	NA	• RDW is a low-cost parameter and can be used for assessing the risk of adverse clinical progression; • Further studies recommended to analyze if RDW is also useful to predict the post-recovery course of COVID-19.
12.	Moutchia et al., 2020	• Sample size ranged from 5 to 1582; Mean/median age: 35 to 68 years; Males: 33.3% to 81%.	NA	• Patients had varied degrees of comorbidities: hypertension, diabetes and cancer	• Severe or critical COVID-19 patients displayed significantly higher counts/levels of WBCs, neutrophils, CRP, IL-6, ESR, ALT, AST, serum creatinine, D-dimer and LDH in comparison to non-severe COVID-19 patients	NA	NA	• Severe COVID-19 displays increased levels of biomarkers of innate immune response, tissue damage and major organ failure; and decreased levels of biomarkers of adaptive immune response.
13.	Jahrami et al., 2021	• Total patients analyzed: 54231; Study population ranged from general public to healthcare workers; Age range: 18 to 60 years; Males: 0% to 91.5%.	• Global pooled prevalence rate of sleep problems among all populations: 35.7% (95% CI 29.4–42.4); • COVID-19 patients most affected with a pooled rate: 74.8% (95% CI 28.7–95.6); • Health care workers and general population: comparable rates: 36.0% (95% CI 21.1–54.2) and 32.3% (95% CI 25.3–40.2), respectively.	NA	NA	NA	NA	• High prevalence (40%) of sleep problems in patients and health-workers; • Sleep self-assessment questionnaires, like, PSQI (39.6%; 95% CI 29.6– 50.6) more sensitive to diagnose sleep problems associated with COVID-19; • Further longitudinal studies required to understand trajectories of sleep problems post-COVID in different populations.
14.	Mudatsir et al., 2020	• Sample size of severe patients ranged from 7 to 926, while that of mildly ill patients ranged from 10 to 283.	• Lower risk of severe COVID-19 due to dry cough vs. productive cough (OR= 0.66, 95% CI 0.44- 0.97) • Higher risk of severe COVID-19 due to: Dyspnea (OR= 3.28, 95% CI 2.09- 5.15), fatigue (OR= 2.00, 95% CI 1.25- 3.20), anorexia (OR= 1.83, 95% CI 1.00- 3.34), elevated respiratory rate (OR= 2.85, 95% CI 1.28- 6.33), dizziness (OR= 2.67, 95% CI 1.18, 6.01), and increased systolic blood pressure (OR: 1.84, 95% CI 1.31- 2.60).	• Higher risk of developing severe form of COVID-19 due to: Chronic respiratory disease (OR= 2.48, 95% CI 1.44- 4.27), cardiovascular disease (OR= 1.70, 95% CI 1.05- 2.78), diabetes mellitus (OR= 2.10, 95% CI 1.33- 3.34), and hypertension (OR= 2.33, 95% CI 1.42- 3.81) were associated with a greater risk of severe COVID-19	• Lower risk of severe COVID-19: low leukocyte levels (OR= 0.59, 95% CI 0.41- 0.87) and elevated lymphocyte (OR= 0.34, 95% CI 0.23- 0.50); • Higher risk of severe COVID-19 indicators: Elevated WBC count (OR= 4.92, 95% CI 2.12- 11.31), raised neutrophil count (OR= 5.45, 95% CI 2.04- 14.54), lymphocytopenia (OR= 3.19, 95% CI 1.14-7.07), reduced hemoglobin levels (OR= 0.76, 95%CI 0.58- 1.00), elevated AST (OR= 4.91), elevated ALT (OR= 3.23), raised SCr (OR= 2.14), elevated BUN (OR= 6.15, 95% CI 3.05- 12.37), elevated Hs-troponin I (OR= 9.25, 95% CI 3.51- 24.37), raised CK (OR= 2.44, 95% CI 1.65- 3.62), high Hs-CRP (OR= 14.27, 95% CI 5.13- 39.71), high IL-6 (OR= 6.68, 95% CI 3.20- 13.94), raised D-dimer (OR= 6.19, 95% CI 4.22- 9.08), increased ferritin (OR= 1.96, 95% CI 1.06- 3.62), high LDH (OR= 8.28, 95% CI 4.75- 14.46), elevated PCT (OR= 6.62, 95% CI 3.32- 13.21), raised ESR (OR= 4.45, 95% CI 2.56- 7.76), and CRP >8 (OR= 8.34, 95% CI 1.85- 37.62).	NA	NA	• COVID-19 exhibits an unknown pattern of disease development; • 34 factors associated with severe COVID-19 were identified in the systematic review and meta-analysis; • These factors may improve the understanding of the disease and allow upgradation of prediction models to enable better prognosis of COVID-19.
15.	Mesas et al., 2020	• Mean age of participants: 40 to 73 years; • Of the 51,225 patients, 24.3% were non-survivors.	• Indicators of mortality: Dyspnea (p-OR= 2.5) and smoking (p-OR= 1.6); • Lower risk of mortality: Headache (p-OR= 0.5), cough (p-OR= 0.7), vomiting (p-OR= 0.6), diarrhea (p-OR= 0.6) and fever (p-OR= 0.8).	• Kidney disease, CVD, Hypertension, Diabetes, Malignancy, Pulmonary disease.	• Decreased albumin and lymphocytes, increased CRP, BUN, IL-6, LDH, neutrophil, Ferritin, Cardiac TnI.	NA	NA	• Epidemiological data should be stratified by age, gender and baseline comorbidities for accurate determination of mortality predictors.
16.	Izcovich et al., 2020	• Total patients: 75607 with range of 10 to 8910 patients per study; • Increasing age identified as risk factor of poor prognosis and mortality.	• Prognostic factors of severity: Hemoptysis (OR= 4.39, 95% CI 2.18- 8.81), abdominal pain (OR= 1.95, 95% CI 1.36- 1.79), fatigue (OR= 1.41, 95% CI 1.19- 1.68), fever (OR= 1.84, 95% CI 1.54- 2.21) and myalgia or arthralgia (OR= 1.29, 95% CI 1.03- 1.61); • Prognostic factors of mortality: Respiratory failure (OR= 21.17, 95% CI 4.9- 91.3), low blood pressure (OR= 6.7, 95% CI 3.14- 14.33), hypoxemia (OR= 5.46, 95% CI 2.05- 14.53), tachycardia (OR= 2.61, 95% CI 1.62- 4.22), dyspnea (OR= 3.45, 95% CI 2.72- 4.38), anorexia (OR= 2.16, 95% CI 1.14- 4.12) and tachypnea (OR= 1.21, 95% CI 1.12- 1.31).	• Indicators of mortality: COPD (OR= 2.43, 95% CI 1.88- 3.14), CKD (OR= 2.27, 95% CI 1.69- 3.05), cerebrovascular disease (OR= 2.85, 95% CI 2.02- 4.01, CVD (OR= 2.12, 95% CI 1.77- 2.56), cardiac arrhythmia (OR= 2.13, 95% CI 1.72- 2.65), arterial hypertension (OR= 2.02, 95% CI 1.71- 2.38), diabetes (OR= 1.84, 95% CI 1.61- 2.1), dementia (OR= 1.54, 95% CI 1.31- 1.81), obesity (OR= 1.41, 95% CI 1.15- 1.74), cancer (OR= 1.35, 95% CI 1.17- 1.55) and dyslipidemia (OR= 1.26, 95% CI 1.06–1.5).	• Severity indicators: High neutrophil count (OR= 5.66, 95% CI 3.71- 8.63), high BNP (OR= 4.99, 95% CI 3.2- 7.77), High BUN (OR= 3.66, 95% CI 2.82- 4.74), high CK (OR= 3.1, 95% CI 2.32- 4.16), high bilirubin (OR= 2.94, 95% CI 2.18- 3.97), high IL-6 (OR= 7.36, 95% CI 2.97- 18.27), elevated ESR (OR= 3.08, 95% CI 2.04- 4.65); • Mortality indicators: High procalcitonin (OR= 12.42, 95% CI 7.18- 21.5), myocardial injury markers (OR= 10.89, 95% CI 5.39- 22.04), high WBC counts (OR= 4.06, 95% CI 2.7- 6.12), high lactate (OR= 3.66, 95% CI 2.26- 5.94), low platelet count (OR= 5.43, 95% CI 2.55- 11.56), high D-dimer (OR= 4.81, 95% CI 3.15- 7.34), high LDH (OR= 4.09, 95% CI 1.18- 14.17), high CRP (OR= 6.6, 95% CI 3.36- 12.99), reduced lymphocyte counts (OR= 3.57, 95% CI 2- 6.67), elevated AST (OR= 3.5, 95% CI 1.59–7.71), elevated albumin levels (OR= 1.53, 95% CI 1.32- 1.78) and increased creatinine (OR= 1.14, 95%CI 1.02- 1.28).	• Severity indicators: Consolidative infiltrate (OR= 2.46, 95% CI 1.54- 3.93) and pleural effusion (OR= 3.31, 95% CI 2.03- 5.38).	NA	• Risk of severe disease and mortality is higher in elderly patients, with previous comorbidities, raised lab biomarkers of inflammation; • Radiological features were not good predictors.
17.	Del Zompo et al., 2020	• Pre-known liver disease in 0% to 37.6% patients; • 46.9% prevalence of at least one abnormal LFT at admission (95% CI 37-56.8, 2306 patients).	NA	• Presence of liver abnormality.	• Elevated AST, ALT, tBIL levels.	NA	NA	• Abnormal LFT findings were considered as hallmark of COVID-19, with association with disease severity and in-hospital mortality.
18.	Hannum et al., 2020	• Sample size ranged from 15 to 7178; Number of smell loss cases/article: 2 to 4668 (prevalence rate: 5% to 98.3%).	• Loss of smell- overall prevalence rate of 50.2%, 95% CI 38.9- 61.5; prevalence rate per study: 5- 88%; • Meta-analysis for pooled prevalence yielded Cochrane’s Q= 5784.14, df= 33, p< 0.001, I^2 ^= 99.4%.	NA	NA	NA	NA	• Longitudinal assessments of chemosensory function would be useful to identify patients with continued impairment who might require further treatment and olfactory training.
19.	Israfil et al., 2021	• Mean age: 50.6 years (range 0.5– 94 years); • Higher proportion of male patients: 60.3% (6567/10889) while female: 39.7% (4322/10889); • Ethnic origin: Asian, European and North American; • Smokers (active): about 14.2% (641/4530); • Severe patients: 37.4% (2408/6446).	• Most common symptoms: Cough/dry cough 59.6% (2146/3598), fever 46.9% (4342/9242), fatigue 27.8% (1000/3598), dyspnea/shortness of breath 20.23% (728/3598), muscle ache/myalgia 12.64% (455/3598), diarrhea 11.95% (430/3598), headache 10.8% (389/3598), anorexia 9.9% (356/3598), sore throat 7.5% (270/3598), expectoration 7.48% (269/3598), upper airway congestion 6.67% (240/3598) and rhinitis 5.86% (211/3598); • Asymptomatic case: 0.56% (20/3598).	• Most common: Hypertension 35.9% (3909/10889), diabetes 20.17% (2196/10889), obesity 15.95% (1735/10889), cardiovascular disease 13.92% (1516/10889), asthma 4.42% (481/10889), COPD 4.31% (469/10889) and malignancy 3.99% (435/10889).	• Most common: lymphocytopenia 55.9% (4177/7470); • Other major findings: Elevated levels of CRP 61.9% (830/1340), AST 53.3% (3481/6537), ALT 35.64% (2318/6503), LDH 40.8% (392/973), ESR 72.99% (173/237), serum ferritin 63% (62/99), (IL-6) 52% (51/99), prothrombin time 35.47% (102/286) and D-dimer 28.06% (179/638).	• Most common abnormality: Bilateral lungs 71.1% (1581/2223); • Other major findings: GGO 48% (432/900), consolidation 21.88% (140/640), pleural effusion 20.6% (195/947), lung lesions 78.3% (180/230), enlarged lymph nodes 50.7% (153/302), thickened bronchial walls 30.3% (80/264), thickened lung texture 84.9% (62/73) and thickened interlobular septa 47.1% (80/170).	NA	• Laboratory investigations and CT scan reports with clinical correlation can provide useful information to enable correct diagnosis and better management of COVID-19 patients.
20.	Poly et al., 2021	• Total sample size of studies analyzed ranged from 58 to 177133; males: 43.3% to 80.2%; Mean/median age: 49.1 to 76 years.	• Obesity significantly associated with an increased risk of mortality (p-RR 1.42, 95% CI 1.24–1.63, p< 0.001); • Class III obesity patients observed a greater risk (p-RR= 1.92, 95% CI 1.50– 2.47, p< 0.001, I^2 ^= 31.99).	Risk of mortality assessment in obese COVID-19 patients: Diabetes (p-RR= 1.19 (95% CI 1.07–1.32, p= 0.001), stroke (p-RR= 1.80 (95% CI 0.89– 3.64, p= 0.10), hypertension (p-RR= 1.07, 95% CI 0.92– 1.25, p= 0.35), CKD (p-RR= 1.57, 95% CI 1.57– 1.91, p< 0.001), COPD (p-RR= 1.34, 95% CI 1.18–1.52, p< 0.001).	NA	NA	NA	• Obesity is a risk factor for mortality in COVID-19; • Clinicians must quickly start medical interventions in obese COVID-19 patients; • Further investigations would urgently be required to understand the pathophysiological association between obesity and risk of COVID-19 related mortality.

NA- Data under the respective heading was either not analyzed or not reported for the entire number of patients in the respective article. ALT, alanine aminotransferase; AST, aspartate aminotransferase; BMI, body mass index; BNP, B-type natriuretic peptide; BUN, blood urea nitrogen; CBC, complete blood count; CI, confidence interval; CK, creatine kinase; CKD, chronic kidney disease; CK-MB, creatine kinase,myocardial band, an isoenzyme of creatine kinase; COPD, chronic obstructive pulmonary disease; COVID-19, coronavirus disease 2019; CRP, C-reactive protein; CT, computed tomography; CVD, cardiovascular disease; df, degree of freedom; ECMO, extracorporeal membrane oxygenation; ESR, erythrocyte sedimentation rate; GGO, ground glass opacity; ICU, intensive care unit; IL (interleukin); KFT, kidney function test; LDH, lactate dehydrogenase; LFT, liver function test; MERS, middle east respiratory syndrome; NT-proBNP, N-terminal pro-BNP; OR, odds ratio; PCT, procalcitonin; p-OR, pooled odds ratio; p-RR, pooled relative risk; PSQI, Pittsburgh sleep quality index; RBC, red blood cell; RDW, RBC distribution width; RDW-CV, RBC distribution width- coefficient of variation; RR, relative risk; SARS, severe acute respiratory syndrome; SCr, serum creatinine; TnI, troponin I; WBC, white blood cell.

**Table 2:** Putative independent predictors of COVID-19 adverse prognosis, severity or mortality.

S. No.	Biomarkers	Article reference	Clinical outcome	Number of patients	Severity or Fatality estimate
**PARAMETERS OF LIVER FUNCTION**					
1.	Elevated AST	Del Zompo et al., 2020	Severe COVID-19	6263	OR= 3.17, 95% CI 2.10 - 4.77
			In-hospital fatality	2395	OR= 4.39, 95% CI 2.68 - 7.18
		Moutchia et al., 2020	Severe/critical COVID-19	2705	MPR= 2.14, 95% CI 1.80 - 2.54
		Jutzeler et al., 2020	Severe COVID-19	184	SMD: 0.85, 95% CI 0.61-1.09
		Zhang JJY. et al., 2020	ICU admission	2153	p= 0.0040
		Li et al., 2021	ICU admission	479*	OR= 3.26, 95% CI 2.40-4.42, p< 0.001, I^2 ^= 5.3%
		Izcovich et al., 2020	Severe COVID-19	9179	OR= 3.41, 95% CI 2.7– 4.3
			Fatality	2969	OR= 3.5, 95% CI 1.59– 7.71
2.	Elevated ALT	Del Zompo et al., 2020	Severe COVID-19	6249	OR= 1.54, 95% CI 1.17 - 2.03
			In-hospital fatality	2613	OR= 1.48, 95% CI 1.12 - 1.96
		Moutchia et al., 2020	Severe/critical COVID-19	2540	MPR= 1.59, 95% CI 1.36 - 1.87
		Zhang JJY. et al., 2020	ICU admission	2153	p=0.024
3.	Increased tBIL	Del Zompo et al., 2020	Severe COVID-19	5153	OR= 2.32, 95% CI 1.18- 4.58
			In-hospital fatality	2086	OR= 7.75, 95% CI 2.28- 26.40
		Izcovich et al., 2020	Severe COVID-19	5098	OR= 2.94, 95% CI 2.18–3.97
4.	Decreased albumin	Jutzeler et al., 2020	Severe COVID-19	131	SMD= -1.60, 95% CI -2.97- (− 0.24)
			Fatality	110	SMD= − 1.14, 95% CI -1.41 – (− 0.85)
		Lu et al., 2020	Fatality	615	OR= 0.11, 95% CI 0.06– 0.19
		Izcovich et al., 2020	Severe COVID-19	1266	OR= 1.11, 95% CI 1.01– 1.21
			Fatality	336	OR= 1.53, 95% CI 1.32– 1.78
**PARAMETERS OF KIDNEY FUNCTION**					
5.	Elevated SCr	Shao et al., 2020	Severe COVID-19	1968	MD= 7.78 µmol/l, 95% CI 4.43-11.14
			Fatality	2138	MD= 20.19 µmol/l, 95% CI 14.96- 25.42
		Moutchia et al., 2020	Severe/critical COVID-19	2019	MPR= 1.90, 95% CI 1.07- 3.36
		Izcovich et al., 2020	Severe COVID-19	1116	OR= 1.89, 95% CI 0.87– 4.10
			Fatality	1508	OR= 1.14, 95% CI 1.02– 1.28
		Li et al., 2021	ICU admission	479*	OR= 2.14, 95% CI 1.14-4.01, p= 0.018, I^2 ^= 0.0%
6.	Higher BUN	Shao et al., 2020	Severe COVID-19	1445	MD= 2.12 µmol/l, 95% CI 1.74 - 2.50
			Fatality	1458	MD= 4.07 µmol/l, 95% CI 3.33- 4.81
		Lu et al., 2020	Fatality	424	OR= 8.49, 95% CI 5.81–12.40
		Izcovich et al., 2020	Severe COVID-19	3890	OR= 3.66, 95% CI 2.82– 4.74
	Elevated blood urea	Moutchia et al., 2020		624	MPR= 3.63, 95% CI 1.73- 7.65
**HAEMATOLOGICAL PARAMETERS**					
7.	Absolute RDW-CV	Lippi et al., 2020	Severe COVID-19	2,654	Fold increase: 1.05, 95% CI 1.03- 1.08-fold
8.	Low hemoglobin	Jutzeler et al., 2020	Severe COVID-19	342	SMD= − 0.23, 95% CI -0.41- (− 0.06)
9.	Low platelet count	Jutzeler et al., 2020	Severe COVID-19	357	SMD= − 0.57, 95% CI -0.68-(-0.45)
		Lu et al., 2020	Fatality	615	OR= 0.33, 95% CI 0.24–0.44
		Li et al., 2021	ICU admission	479*	OR= 2.82, 95% CI 2.07-3.83, p< 0.001, I^2 ^= 0.0%
		Izcovich et al., 2020	Fatality	3676	OR= 5.43, 95% CI 2.55– 11.56
10.	Low lymphocyte count	Henry et al., 2020	Severe COVID-19	1140	OR= 4.20, 95% CI 3.46-5.09
			In-hospital fatality	800	OR= 3.71, 95% CI 1.63 - 8.44
					Severe lymphopenia
					(< 0.5×10×^9^/l) had 12-fold increased odds of in-hospital mortality.
		Mudatsir et al., 2020	Severe COVID-19	198^$^	OR= 3.19, 1.14- 7.07, p< 0.0001
		Moutchia et al., 2020	Severe COVID-19	3875	MPR= 1.74, 95% CI 1.43, 2.12
		Jutzeler et al., 2020	Fatality	255	SMD = − 0.92, 95% CI -1.3 – (− 0.55)
		Li et al., 2021	ICU admission	479*	OR= 4.60, 95% CI 3.25-6.51, p< 0.001, I^2 ^= 0.0%
		Lu et al., 2020	Fatality	615	OR= 0.21, 95% CI 0.12– 0.38
		Izcovich et al., 2020	Severe COVID-19	1909	OR= 2.28, 95% CI 1.21– 4.30
			Fatality	544	OR= 3.57, 95% CI 2– 6.67
11.	Increased neutrophil count	Henry et al., 2020	Severe COVID-19	313	OR= 7.99, 95% CI 1.77- 36.14
			Fatality	183	OR= 7.87, 95% CI 1.75- 35.35
		Moutchia et al., 2020	Severe COVID-19	1237	Severe COVID-19 with higher likelihood
					MPR= 4.29, 95% CI 1.74 - 10.64
		Lu et al., 2020	Fatality	274	OR= 17.56, 95% CI 10.67–28.90
		Izcovich et al., 2020	Severe COVID-19	4945	OR= 5.66, 95% CI 3.71–8.63
12.	Increased leukocyte count	Moutchia et al., 2020	Severe COVID-19	3455	Severe COVID-19 with higher likelihood
					MPR= 3.95, 95% CI 2.35- 6.65
		Jutzeler et al., 2020	Fatality	277	SMD = 2.21, 95% CI 0.61–3.64
		Lu et al., 2020	Fatality	615	OR= 9.13, 95% CI 5.71–14.59
		Zhang JJY. et al., 2020	ICU admission	2153	p< 0.0001
			Fatality		p= 0.0005
		Izcovich et al., 2020	Severe COVID-19	9331	OR= 4.67, 95% CI 3.17– 6.88
			Fatality	2870	OR= 4.06, 95% CI 2.7– 6.12
		Mudatsir et al., 2020	Severe COVID-19	102^$^	OR= 5.38, 95% CI 3.36- 8.62
**PARAMETERS OF INFLAMMATION/INFECTION**					
13.	Elevated CRP	Moutchia et al., 2020	Severe/critical COVID-19	2740	MPR= 1.60, 95% CI 1.32- 1.93
		Jutzeler et al., 2020	Severe COVID-19	277	SMD= 1.47, 95% CI 0.88–2.07
		Lu et al., 2020	Fatality	424	OR= 12.11, 95% CI 5.24–27.98
		Izcovich et al., 2020	Severe COVID-19	9094	OR= 4.5, 95% CI 3.1– 6.23
			Fatality	2107	OR= 6.6, 95% CI 3.36– 12.99
		Li et al., 2021	ICU admission	479*	OR= 4.02, 95% CI 2.80-5.79, p≤ 0.001, I^2 ^= 11.1%
14.	Higher ESR	Moutchia et al., 2020	Severe/critical COVID-19	545	MPR= 1.67, 95% CI 0.67- 4.17
15.	Increased IL-6	Jutzeler et al., 2020	Fatality	110	SMD= 1.21, 95% CI 0.93–1.5
		Izcovich et al., 2020	Severe COVID-19	1211	OR= 7.36, 95% CI 2.97- 18.27
16.	Elevated PCT	Zhang JJY. et al., 2020	ICU admission	2153	p< 0.0001
		Li et al., 2021	ICU admission	479*	OR= 6.69, 95% CI 3.99-11.20, p≤ 0.001, I^2 ^= 13.6%
		Izcovich et al., 2020	Fatality	4735	OR= 12.42, 95% CI 7.18– 21.5
**OTHER BIOCHEMICAL PARAMETER**					
17.	Elevated serum ferritin	Moutchia et al., 2020	Severe/critical COVID-19	412	MPR= 2.3, 95% CI 1.67- 3.17
**COAGULATION PARAMETER**					
18.	Elevated D-dimer	Moutchia et al., 2020	Severe/critical COVID-19	2030	MPR= 2.27, 95% CI 1.67- 3.09
		Figliozzi et al., 2020	Combined adverse outcome (ICU admission or IMV or fatality)	3270	OR= 4.39, 95% CI 1.85-10.41, p= 0.003
			Fatality		OR= 4.40, 95% CI 1.10-17.58, p= 0.04
		Izcovich et al., 2020	Severe COVID-19	6356	OR= 3.27, 95% CI 2.46– 4.36
			Fatality	4361	OR= 4.81, 95% CI 3.15–7.34
19.	Prolonged PT	Jutzeler et al., 2020	Fatality	206	SMD = 7.99, 95% CI 4.64–11.34
**MARKER OF TISSUE DAMAGE**					
20.	Elevated LDH	Moutchia et al., 2020	Severe/critical COVID-19	1893	MPR= 2.41, 95% CI 1.65- 3.51
		Jutzeler et al., 2020	Severe COVID-19	93	SMD = 1.71, 95% CI 1.08–2.34
		Lu et al., 2020	Fatality	465	OR= 37.52, 95% CI 24.68–57.03
		Zhang JJY. et al., 2020	ICU admission	2153	p< 0.0001
			ARDS		p< 0.0001
			Fatality		p< 0.0001
		Izcovich et al., 2020	Severe COVID-19	7955	OR= 4.48, 95% CI 3.21–6.25
			Fatality	1440	OR= 4.09, 95% CI 1.18– 14.17
**PARAMETERS OF CARDIAC INJURY**					
21.	Increased TnI or myocardial injury	Izcovich et al., 2020	Severe COVID-19	3627	OR= 10, 95% CI 6.84–14.62
			Fatality	3855	OR= 10.89, 95% CI 5.39–22.04
	Elevated TnI or TnT	Li et al., 2020	Fatality	1028	RR= 4.69, 95% CI 3.39- 6.48, p< 0.001, I^2 ^= 22.5%
22.	Elevated CK	Li et al., 2020	Severe COVID-19	2174	RR= 1.98, 95% CI 1.50- 2.61, p< 0.001, I^2 ^= 0.0%

NA- The parameter value was not applicable or not mentioned or could not be ascertained with the given information. *- The number of patients as mentioned for ICU COVID-19 cases in overall results section. $- The number of severe and non-severe patients as per the table in the citation. May or may not be specific to the respective parameter. ALT, alanine aminotransferase; ARDS, acute respiratory distress syndrome; AST, aspartate aminotransferase; BUN, blood urea nitrogen; CRP, C-reactive protein; CK, creatine kinase; ESR, erythrocyte sedimentation rate; LDH, lactate dehydrogenase; MD, mean difference; MPR, meta-prevalence ratio; SMD, standardized mean difference; NA, not applicable; OR, odds ratio; PCT, procalcitonin; PT, prothrombin time; RDW-CV, red blood cell distribution width-coefficient of variation; RR, relative risk; SCr, serum creatinine; tBIL, total bilirubin; TnI, troponin I; TnT, troponin T.

A systematic review and meta-analysis of 36 studies involving more than 20000 patients demonstrated important findings (Del Zompo et al., 2020). With an intent to correlate liver injury with clinical outcomes in COVID-19 patients, the researchers found that nearly 47% of COVID-19 cases had abnormal LFT. They also found that the laboratory tested AST, ALT and total bilirubin were independent predictors of COVID-19 severity and in-hospital mortality (**Table 2**) (Del Zompo et al., 2020). However, there was insufficient information on the etiology of pre-existing liver injury in COVID-19 patients at the time of hospitalization. Hence, further prospective cohort studies would be essential to validate these findings.

Other noteworthy biochemical findings are elevated levels of BUN and serum creatinine (SCr) (Shao et al., 2020). A robust meta-analysis recorded significant (p< 0.00001) rise in levels of BUN and SCr in severe COVID-19 cases and non-survivors (**Table 1B**) (Shao et al., 2020). Increased SCr and BUN values were identified as independent biomarkers for COVID-19 related severity and in-hospital mortality early during the pandemic (Chen et al., 2020; Cheng et al., 2020). However, the rate of severe and fatal cases in the study by Shao et al. was quite high, which could be due to the fact that the studies analyzed represented majorly poor COVID-19 outcomes (Shao et al., 2020). Hence, over-estimation of severity and fatality rate may be a limitation to this otherwise crucial set of findings.

### Radiological Investigations

Identification of viral pathogens is possible by careful examination of imaging patterns since the latter are associated with viral pathogenesis. Since, viruses belonging to a single viral family share a similar pathogenesis, computed tomography (CT) was considered a trusted technique to distinguish patterns and features of COVID-19 in immunocompetent patients (Chung et al., 2020; Jin et al., 2020; Tao et al., 2020). The systematic review and meta-analysis conducted by Jutzeler et al. identified abnormal CT scans in nearly 90% (89.6% specifically) of the COVID-19 confirmed adult patients, 88.6% of the pregnant patients and 65% of the pediatric and neonatal patients (Jutzeler et al., 2020). The major finding in CT imaging was the occurrence of unilateral or bilateral pneumonia in 83.6% (6620/7917) adult patients, 88.6% (31/35) pregnant and 65% (194/298) pediatric and neonatal COVID-19 cases (Jutzeler et al., 2020). Other prominent abnormal CT features included air bronchogram (50.5%, 264/523) and ground-glass opacity (GGO) with consolidation (47.4%, 153/323) and without (43.8%, 2446/5591) in adult patients, GGO (85.3%, 29/34) and consolidation (50%, 8/16) in pregnant patients, and GGO (38.9%, 108/278) and local patchy shadowing (23.3%, 52/223) in pediatric and neonatal patients (Jutzeler et al., 2020).

## Conclusions

Identification of high-risk clinical and laboratory features contribute to early prediction, diagnosis and efficient treatment of patients (Li et al., 2021). A fatality rate of 7.7% with about 8% of the COVID-19 patients being asymptomatic was observed during the early pandemic period (Jutzeler et al., 2020). Since, it is difficult to record the exact number of asymptomatic cases, owing to obvious reasons (like no hospital/clinic visit, hence no medical record; or lack of awareness that a potentially fatal disease can be asymptomatic in some patients) such value is deemed to be 6- to 10- fold higher (Jutzeler et al., 2020). Hence, more aggressive antigen detection, as well as serological surveillance of contacts of confirmed COVID-19 patients, is necessary to enable screening and identification of asymptomatic COVID-19 patients. Further, prospective well-planned cohort studies would be necessary to enable further characterization of the overall, gender-specific and/or geographical location-based risk factors.

It is imperative to categorize COVID-19 patients based on their comorbidities, like impaired kidney or liver functions or cardiac injury, etc. As discussed in the present work, AKI is a critical complication of COVID-19 and calls for immediate care and monitoring (Shao et al., 2020) to minimize the risk of severity and poor prognosis. Similarly, abnormal LFTs are important early predictors of COVID-19 severity and in-hospital mortality (Del Zompo et al., 2020). Also, pre-existing chronic liver disease, especially cirrhosis, is an indicator of a high risk of mortality. Hence, aggressive interventions for such cases must be exercised. This would enable better patient management and may improve the disease outcome. Measurement of anthropometric parameters, especially BMI, is also recommended for COVID-19 patient management, importantly for patients who are or above 65 years of age (Földi et al., 2020; Poly et al., 2021). Basic hematological screening that can be done with minimal resources can be a life-saver. The findings that lymphopenia and neutrophilia at the time of hospital admission indicate poor COVID-19 outcome call for routine hematological monitoring. It would enable an early careful intervention in such patients enabling better patient care. Such regular monitoring may also aid in the stratification and the management of risk associated with COVID-19 (Henry et al., 2020). Further, it is also important to stratify epidemiological data based on demographic characteristics and risk factors for adverse COVID-19 outcomes, to enable exact and aggressive patient care (Mesas et al., 2020). Based on the analysis in this work, we can conclude that careful monitoring of clinical data, risk factors and disease biomarkers (Israfil et al., 2021) may enable early determination of COVID-19-led severity. Such an early estimate would be helpful in efficient patient management and possibly minimize the related mortality.

## Author Contributions

JS conceptualized the study, retrieved the articles, analyzed the data and guided inclusion of specific information, drafted and proof-read the manuscript. RR reviewed the data, analyzed the information, tabulated findings, drafted and proof-read the manuscript. MB helped in information retrieval and inclusion of findings. PA provided intellectual inputs and proof-read the manuscript. VS conceived the study, provided intellectual inputs, guided the inclusion of information, proof-read and approved the final version of the manuscript. All authors contributed to the article and approved the submitted version.

## Funding

JS received institutional support from AIIMS, Bathinda, Punjab, India. RR is presently an independent research fellow (Research Associate) of the Council of Scientific and Industrial Research (CSIR), Government of India. VS received financial support (Faculty Recharge Programme) from the University Grants Commission (UGC), Govt. of India.

## Conflict of Interest

The authors declare that the research was conducted in the absence of any commercial or financial relationships that could be construed as a potential conflict of interest.

## Publisher’s Note

All claims expressed in this article are solely those of the authors and do not necessarily represent those of their affiliated organizations, or those of the publisher, the editors and the reviewers. Any product that may be evaluated in this article, or claim that may be made by its manufacturer, is not guaranteed or endorsed by the publisher.

## References

[B1] Coronavirus Could Cost the Global Economy Trillions on a Sars Baseline. Available at: https://www.ccn.com/coronavirus-could-cost-the-global-economy-trillions-on-a-sars-baseline/.

[B2] Cheng Y. Luo R. Wang K. Zhang M. Wang Z. Dong L.. (2020). Kidney Disease Is Associated With in-Hospital Death of Patients With COVID-19. Kidney Int. 97 (5), 829–838. doi: 10.1016/j.kint.2020.03.005 32247631 PMC7110296

[B3] Chen N. Zhou M. Dong X. Qu J. Gong F. Han Y.. (2020). Epidemiological and Clinical Characteristics of 99 Cases of 2019 Novel Coronavirus Pneumonia in Wuhan, China: A Descriptive Study. Lancet 395 (10223), 507–513. doi: 10.1016/s0140-6736(20)30211-30217 32007143 PMC7135076

[B4] Chung M. Bernheim A. Mei X. Zhang N. Huang M. Zeng X.. (2020). CT Imaging Features of 2019 Novel Coronavirus, (2019-Ncov). Radiology 295 (1), 202–207. doi: 10.1148/radiol.2020200230 32017661 PMC7194022

[B5] Del Zompo F. De Siena M. Ianiro G. Gasbarrini A. Pompili M. Ponziani F. R. (2020). Prevalence of Liver Injury and Correlation With Clinical Outcomes in Patients With COVID-19: Systematic Review With Meta-Analysis. Eur. Rev. Med. Pharmacol. Sci. 24 (24), 13072–13088. doi: 10.26355/eurrev_202012_24215 33378061

[B6] Emem M. (2020) Multi-Billion Dollar Coronavirus Vaccine Deal Could Save the Economy. CCN Headlines Opinion. Available at: https://www.ccn.com/coronavirus-could-cost-the-global-economy-trillions-on-a-sars-baseline/.

[B7] Ferrando M. Bagnasco D. Roustan V. Canonica G. W. Braido F. Baiardini I. (2016). Sleep Complaints and Sleep Breathing Disorders in Upper and Lower Obstructive Lung Diseases. J. Thorac. Dis. 8 (8), E716–E725. doi: 10.21037/jtd.2016.07.82 27621908 PMC4999711

[B8] Figliozzi S. Masci P. G. Ahmadi N. Tondi L. Koutli E. Aimo A.. (2020). Predictors of Adverse Prognosis in COVID-19: A Systematic Review and Meta-Analysis. Eur. J. Clin. Invest. 50 (10), e13362. doi: 10.1111/eci.13362 32726868

[B9] Földi M. Farkas N. Kiss S. Zádori N. Váncsa S. Szakó L.. (2020). Obesity Is a Risk Factor for Developing Critical Condition in COVID-19 Patients: A Systematic Review and Meta-Analysis. Obes. Rev. 21 (10), e13095. doi: 10.1111/obr.13095 32686331 PMC7404429

[B10] Ghahramani S. Tabrizi R. Lankarani K. B. Kashani S. M. A. Rezaei S. Zeidi N.. (2020). Laboratory Features of Severe vs. non-Severe COVID-19 Patients in Asian Populations: A Systematic Review and Meta-Analysis. Eur. J. Med. Res. 25 (1), 30. doi: 10.1186/s40001-020-00432-3 32746929 PMC7396942

[B11] Gu X. Li X. An X. Yang S. Wu S. Yang X.. (2020). Elevated Serum Aspartate Aminotransferase Level Identifies Patients With Coronavirus Disease 2019 and Predicts the Length of Hospital Stay. J. Clin. Lab. Anal. 34, e23391. doi: 10.1002/jcla.23391 32488888 PMC7300531

[B12] Hannum M. E. Ramirez V. A. Lipson S. J. Herriman R. D. Toskala A. K. Lin C.. (2020). Objective Sensory Testing Methods Reveal a Higher Prevalence of Olfactory Loss in COVID-19 Positive Patients Compared to Subjective Methods: A Systematic Review and Meta-Analysis. Chem. Senses. 45 (9), 865–874. doi: 10.1093/chemse/bjaa064 33245136 PMC7543258

[B13] Henry B. Cheruiyot I. Vikse J. Mutua V. Kipkorir V. Benoit J.. (2020). Lymphopenia and Neutrophilia at Admission Predicts Severity and Mortality in Patients With COVID-19: A Meta-Analysis. Acta BioMed. 91 (3), e2020008. doi: 10.23750/abm.v91i3.10217 PMC7716963 32921706

[B14] Huang C. Wang Y. Li X. Ren L. Zhao J. Hu Y.. (2020). Clinical Features of Patients Infected With 2019 Novel Coronavirus in Wuhan, China. Lancet 395, 497–506. doi: 10.1016/S0140-6736(20)30183-5 31986264 PMC7159299

[B15] Israfil S. M. H. Sarker M. M. R. Rashid P. T. Talukder A. A. Kawsar K. A. Khan F.. (2021). Clinical Characteristics and Diagnostic Challenges of COVID-19: An Update From the Global Perspective. Front. Public Health 8, 567395. doi: 10.3389/fpubh.2020.567395 33505949 PMC7831046

[B16] Izcovich A. Ragusa M. A. Tortosa F. Lavena Marzio M. A. Agnoletti C. Bengolea A.. (2020). Prognostic Factors for Severity and Mortality in Patients Infected With COVID-19: A Systematic Review. PloS One 15 (11), e0241955. doi: 10.1371/journal.pone.0241955 33201896 PMC7671522

[B17] Jahrami H. BaHammam A. S. Bragazzi N. L. Saif Z. Faris M. Vitiello M. V. (2021). Sleep Problems During the COVID-19 Pandemic by Population: A Systematic Review and Meta-Analysis. J. Clin. Sleep Med. 17 (2), 299–313. doi: 10.5664/jcsm.8930 33108269 PMC7853219

[B18] Jin Y.-H. Cai L. Cheng Z.-S. Cheng H. Deng T. Fan Y.-P.. (2020). A Rapid Advice Guideline for the Diagnosis and Treatment of 2019 Novel Coronaviru-Ncov) Infected Pneumonia (Standard Version). Mil. Med. Res. 7 (1), 4. doi: 10.1186/s40779-020-0233-6 32029004 PMC7003341

[B19] Jutzeler C. R. Bourguignon L. Weis C. V. Tong B. Wong C. Rieck B.. (2020). Comorbidities, Clinical Signs and Symptoms, Laboratory Findings, Imaging Features, Treatment Strategies, and Outcomes in Adult and Pediatric Patients With COVID-19: A Systematic Review and Meta-Analysis. Travel Med. Infect. Dis. 37, 101825. doi: 10.1016/j.tmaid.2020.101825 32763496 PMC7402237

[B20] Li J. He X. Yuan Y. Zhang W. Li X. Zhang Y.. (2021). Meta-Analysis Investigating the Relationship Between Clinical Features, Outcomes, and Severity of Severe Acute Respiratory Syndrome Coronavirus 2 (SARS-Cov-2) Pneumonia. Am. J. Infect. Control 49 (1), 82–89. doi: 10.1016/j.ajic.2020.06.008 32540370 PMC7292004

[B21] Li X. Pan X. Li Y. An N. Xing Y. Yang F.. (2020). Cardiac Injury Associated With Severe Disease or ICU Admission and Death in Hospitalized Patients With COVID-19: A Meta-Analysis and Systematic Review. Crit. Care 24, 468. doi: 10.1186/s13054-020-03183-z 32723362 PMC7386170

[B22] Lippi G. Henry B. M. Sanchis-Gomar F. (2020). Red Blood Cell Distribution Is a Significant Predictor of Severe Illness in Coronavirus Disease 2019. Acta Haematol. 25, 1–5. doi: 10.1159/000510914 PMC7490490 32841949

[B23] Loomba R. S. Aggarwal G. Aggarwal S. Flores S. Villarreal E. G. Farias J. S.. (2021). Disparities in Case Frequency and Mortality of Coronavirus Disease 2019 (COVID-19) Among Various States in the United States. Ann. Med. 53 (1), 151–159. doi: 10.1080/07853890.2020.1840620 33138653 PMC7877922

[B24] Lu L. Zhong W. Bian Z. Li Z. Zhang K. Liang B.. (2020). A Comparison of Mortality-Related Risk Factors of COVID-19, SARS, and MERS: A Systematic Review and Meta-Analysis. J. Infect. 81 (4), e18–e25. doi: 10.1016/j.jinf.2020.07.002 PMC7334925 32634459

[B25] Malaiyan J. Arumugam S. Mohan K. Radhakrishnan G. (2021). An Update on the Origin of SARS-Cov-2: Despite Closest Identity, Bat (Ratg13) and Pangolin Derived Coronaviruses Varied in the Critical Binding Site and O-Linked Glycan Residues. J. Med. Virol. 93 (1), 499–505. doi: 10.1002/jmv.26261 32633815 PMC7361880

[B26] Mesas A. E. Cavero-Redondo I. Álvarez-Bueno C. Sarriá Cabrera M. A. Maffei de Andrade S. Sequí-Dominguez I.. (2020). Predictors of in-Hospital COVID-19 Mortality: A Comprehensive Systematic Review and Meta-Analysis Exploring Differences by Age, Sex and Health Conditions. PloS One 15 (11), e0241742. doi: 10.1371/journal.pone.0241742 33141836 PMC7608886

[B27] Moutchia J. Pokharel P. Kerri A. McGaw K. Uchai S. Nji M.. (2020). Clinical Laboratory Parameters Associated With Severe or Critical Novel Coronavirus Disease 2019 (COVID-19): A Systematic Review and Meta-Analysis. PloS One 15 (10), e0239802. doi: 10.1371/journal.pone.0239802 33002041 PMC7529271

[B28] Mudatsir M. Fajar J. K. Wulandari L. Soegiarto G. Ilmawan M. Purnamasari Y.. (2020). Predictors of COVID-19 Severity: A Systematic Review and Meta-Analysis. F1000Res 9, 1107. doi: 10.12688/f1000research.26186.2 33163160 PMC7607482

[B29] Poly T. N. Islam M. M. Yang H. C. Lin M. C. Jian W. S. Hsu M. H.. (2021). Obesity and Mortality Among Patients Diagnosed With COVID-19: A Systematic Review and Meta-Analysis. Front. Med. 8, 620044. doi: 10.3389/fmed.2021.620044 PMC7901910 33634150

[B30] Shao M. Li X. Liu F. Tian T. Luo J. Yang Y. (2020). Acute Kidney Injury Is Associated With Severe Infection and Fatality in Patients With COVID-19: A Systematic Review and Meta-Analysis of 40 Studies and 24,527 Patients. Pharmacol. Res. 161, 105107. doi: 10.1016/j.phrs.2020.105107 32739424 PMC7393179

[B31] Shi Z. L. (2021). Origins of SARS-Cov-2: Focusing on Science. Infect. Dis. Immun. 1 (1), 3–4. doi: 10.1097/ID9.0000000000000008 PMC8057312 38630114

[B32] Shi L. Lu Z.-A. Que J.-Y. Huang X.-L. Lu L. Ran M.-S.. (2020). Prevalence of and Risk Factors Associated With Mental Health Symptoms Among the General Population in China During the Coronavirus Disease 2019 Pandemic. JAMA Netw. Open 3 (7), e2014053. doi: 10.1001/jamanetworkopen.2020.14053 32609353 PMC7330717

[B33] Singh D. P. Jamil R. T. Mahajan K. (2020). “Nocturnal Cough,” in Statpearls. Eds. Cascella M. Rajnik M. Cuomo A. Dulebohn S. C. Di Napoli R. S. (Treasure Island, FL: StatPearls Publishing).

[B34] Song Z. Xu Y. Bao L. Zhang L. Yu P. Qu Y.. (2019). From SARS to MERS, Thrusting Coronaviruses Into the Spotlight. Viruses 11 (1), 59. doi: 10.3390/v11010059 PMC6357155 30646565

[B35] Tao A. Zhenlu Y. Hongyan H. Chenao Z. Chong C. Wenzhi L.. (2020). Correlation of Chest CT and RT-PCR Testing in Coronavirus Disease 2019 (COVID-19) in China: A Report of 1014 Cases. Radiology 296 (2), E32–E40. doi: 10.1148/radiol.2020200642 32101510 PMC7233399

[B36] Undurraga E. A. Chowell G. Mizumoto K. (2021). COVID-19 Case Fatality Risk by Age and Gender in a High Testing Setting in Latin America: Chile, March–August 2020. Infect. Dis. Poverty 10, 11. doi: 10.1186/s40249-020-00785-1 33531085 PMC7854021

[B37] WHO (2021) WHO Coronavirus Disease (COVID-19) Dashboard. Updated on: 27 February 2021. Available at: https://covid19.who.int/ (Accessed on: July 15, 2021).

[B38] WHO News release (2021) WHO Calls for Further Studies, Data on Origin of SARS-Cov-2 Virus, Reiterates That All Hypotheses Remain Open. Available at: https://www.who.int/news/item/30-03-2021-who-calls-for-further-studies-data-on-origin-of-sars-cov-2-virus-reiterates-that-all-hypotheses-remain-open.

[B39] Woo P. C. Lau S. K. Lam C. S. Lau C. C. Y. Tsang A. K. L. Lau J. H. N.. (2012). Discovery of Seven Novel Mammalian and Avian Coronaviruses in the Genus Deltacoronavirus Supports Bat Coronaviruses as the Gene Source of Alphacoronavirus and Betacoronavirus and Avian Coronaviruses as the Gene Source of Gammacoronavirus and Deltacoronavirus. J. Virol. 86, 3995–4008. doi: 10.1128/JVI.06540-11 22278237 PMC3302495

[B40] Zhang J. J. Y. Lee K. S. Ang L. W. Leo Y. S. Young B. E. (2020). Risk Factors for Severe Disease and Efficacy of Treatment in Patients Infected With COVID-19: A Systematic Review, Meta-Analysis, and Meta-Regression Analysis. Clin. Infect. Dis. 71 (16), 2199–2206. doi: 10.1093/cid/ciaa576 32407459 PMC7239203

[B41] Zhang T. Wu Q. Zhang Z. (2020). Probable Pangolin Origin of SARS-Cov-2 Associated With the COVID-19 Outbreak. Curr. Biol. 30 (8), 1578. doi: 10.1016/j.cub.2020.03.063 32315626 PMC7169893

[B42] Zhu Z. Lian X. Su X. Wu W. Marraro G. A. Zeng Y. (2020). From SARS and MERS to COVID-19: A Brief Summary and Comparison of Severe Acute Respiratory Infections Caused by Three Highly Pathogenic Human Coronaviruses. Respir. Res. 21 (1), 224. doi: 10.1186/s12931-020-01479-w 32854739 PMC7450684

